# Hearing Loss in Osteogenesis Imperfecta: Characteristics and Treatment Considerations

**DOI:** 10.4061/2011/983942

**Published:** 2011-12-14

**Authors:** Joseph P. Pillion, David Vernick, Jay Shapiro

**Affiliations:** ^1^Department of Audiology, Kennedy Krieger Institute, Baltimore, MD 21205, USA; ^2^Department of Physical Medicine and Rehabilitation, Johns Hopkins University School of Medicine, Baltimore, MD 21205, USA; ^3^Department of Otology and Laryngology, Harvard Medical School, Boston, MA 02115, USA; ^4^Bone and Osteogenesis Imperfecta Department, Kennedy Krieger Institute, Baltimore, MD 21205, USA

## Abstract

Osteogenesis imperfecta (OI) is the most common heritable disorder of connective tissue. It is associated with fractures following relatively minor injury, blue sclerae, dentinogenesis imperfecta, increased joint mobility, short stature, and hearing loss. Structures in the otic capsule and inner ear share in the histologic features common to other skeletal tissues. OI is due to mutations involving several genes, the most commonly involved are the COL1A1 or COL1A2 genes which are responsible for the synthesis of the proalpha-1 and proalpha-2 polypeptide chains that form the type I collagen triple helix. A genotype/phenotype relationship to hearing loss has not been established in OI. Hearing loss is commonly found in OI with prevalence rates ranging from 50 to 92% in some studies. Hearing loss in OI may be conductive, mixed, or sensorineural and is more common by the second or third decade. Treatment options such as hearing aids, stapes surgery, and cochlear implants are discussed.

## 1. Introduction

Osteogenesis imperfecta (OI) is the most common of the heritable disorders of bone as first defined by McKusick in 1956 [1]. Approximately 25,000 individuals with this condition live in the US. However, the real incidence may be twice that number because of persons with mild OI who experience a small number of fractures and in whom no diagnosis is made. Hearing loss is a significant complication of OI.

The hallmark of OI is the occurrence of fractures with relatively minor injury. Blue sclerae, dentinogenesis imperfecta, increased joint mobility, and short stature are common to the 8 phenotypes that are currently recognized (Table 1). As shown in Table 1, these vary considerably in their relative incidence and clinical severity. It is not commonly appreciated, however, that OI is a systemic disorder of connective tissue due to the wide distribution of type I collagen in multiple organs. 

Type I collagen is the most abundant protein in the body. OI is the result of mutations involving several genes responsible for the synthesis or intracellular processing of type I collagen (COL1). To date, 7 genes have been implicated as causative in OI [2, 3]. Ninety-five percent of OI cases are due to dominantly transmitted mutations involving COL1A1 or COL1A2 genes which are responsible for the synthesis of the proalpha-1 and proalpha-2 polypeptide chains that form the type I collagen triple helix. Approximately 3–5% of cases involve mutations which are transmitted recessively and are associated with severe or lethal OI. These genes modify the intracellular processing of type I collagen: CRTAP (collagen-related protein), P3H1/LEPRE1 (prolyl 3-hydroxylase 1), and PPIB (Cytophyllin B protein) and several chaperone proteins such as HSP-47 in the endoplasmic reticulum [4–6]. CRTAP and P3H1 form a complex with cyclophilin B (CyPB) in the endoplasmic reticulum (ER) which 3-hydroxylates the Pro986 residue of collagen proalpha1 (1) and proalpha (2I) chains [7]. Mutations in these genes lead to severe or lethal skeletal disease (OI types VII and VIII) [8]. No correlation has been established to date between mutations affecting the COL1A1 and COL1A2 genes and hearing loss in OI [9].

As noted above, disordered type I collagen synthesis affects multiple organs in addition to bone. Blue sclerae result from altered light reflectance in the presence of abnormal scleral collagen [10]. Corneal defects occur because collagen is the dominant corneal protein. Tendon and ligament involvement leads to hyperextensible joints. Dentinogenesis imperfecta is the result of abnormal collagen in dental pulp which leads to enamel breakage. Type I collagen in vascular structures leads to mitral and aortic valve involvement as well as aortic dilatation in some cases. Finally, disordered type I collagen in the ear involves each of the auditory structures, both hard and soft tissues, leading to early-onset hearing loss. 

There is limited information about the histopathology of the temporal bone in OI. Berger et al. examined the histopathologic findings in 8 temporal bones from 5 patients with type III osteogenesis imperfecta [11]. Evidence of both deficient and abnormal ossification was found in the bony walls of middle ear and ossicles. Microfractures were found in the otic capsule and in the anterior process and handle the malleus and at the crura of the stapes. The cochlear and vestibular end organs appeared normal. It is of interest that the OI auditory system did not show changes compatible with otosclerosis. Zajtchuk and Lindsay [12], reporting on three OI cases (Type III), observed sclerotic fixation of the footplate, lack of deposition of the skein-like bone in the endochondral layer, sparse bony septae in marrow spaces, and a deficiency of the periosteal layer. The stapedial crura were thin, deformed, and fractured in 2 cases [12]. Examination with the scanning electron microscope using the Ca/P ratio as the criterion based on characteristic X-ray fluorescence showed that the stapes in OI had a higher Ca/P ratio (2.6 : 1) as compared to the normal stapes 2 : 1 ratio. The stapes in OI were poorly mineralized, with low calcium salt and apparent increase of phosphates. This finding suggested a possible change from hydroxyapatite (or apatite) to brushite, which implied an acidification of bone [13].

Computerized tomography (CT) of the temporal bone in an OI type I patient demonstrated otic capsule demineralization that appeared to progress as hearing diminished [8]. Band-like areas of lucency were seen surrounding the cochlea. Similarly, magnetic resonance imaging (MRI) examination of the otic capsule in type I OI demonstrated demineralized pericochlear lesions with soft tissue signal intensity and contrast enhancement [14, 15]. 

## 2. Hearing Loss in Osteogenesis Imperfecta

Hearing loss is a significant clinical feature in many patients with OI. In national surveys of hearing loss in OI, prevalence rates of hearing loss of 46% [16] to 57.9% [17] have been reported. In a recent study, hearing loss was found in 62% of ears with the hearing loss ranging from mild to profound [18]. Slightly lower prevalence of hearing loss (50%) [19, 20] or even a higher prevalence of hearing loss (65–72%) [16, 21] has been reported. Methodological or definitional differences may account for the varying results across studies [22]. Some investigators have excluded cases of conductive hearing loss believed to be secondary to otitis media with effusion (OME) in younger patients with OI [16, 23] which would lead to underestimation of the prevalence of hearing loss. Subject selection issues are also a consideration. Hearing loss varies with the type of OI and is reported to be most common in type I OI and rarely reported in type IV OI [24]. Studies including only type I OI patients [23] have reported a higher prevalence of hearing loss in OI than studies with more inclusive selection criteria [18]. For example, when only patients with type I OI over 30 years of age are considered, a 95% rate of hearing loss has been reported [23]. No relationship has been reported between the degree, nature of hearing loss, and mutated gene or mutation type [25]. Sykes et al. reported that 13/16 patients with early onset of hearing loss had a mutation in the COL1A1 gene [26]. OI may be associated with neurological complications such as skull fractures, seizure disorders, and brainstem compression secondary to basilar invagination [27]. Some of these complications can result in hearing loss. Some studies have excluded patients with these or other complications believed to be unrelated directly to OI [17], whereas others have not [18, 22]. 

It is frequently reported that conductive hearing loss is found in younger patients with OI, whereas sensorineural hearing loss is found more in older patients with OI [17, 19, 21, 23, 28]. This view is not uniform as one study has reported that hearing loss in OI is most frequently sensorineural in origin [29] in the initial presentation. However, it should be noted that there are definitional differences across studies in terms of how conductive and sensorineural hearing loss are defined. For example, Riedner et al. [19] defined as conductive any hearing loss in which the air-bone gap was 5 dB or greater at two or more octave frequencies from 500 to 4000 Hz. On the other hand, Shapiro et al. [29] required a 15 dB or greater air-bone gap at one or more frequencies to classify a loss as conductive. Other studies have not explained how conductive versus sensorineural hearing loss was defined [28].

 Several investigators have reported that hearing loss is infrequent in OI until the 2nd or 3rd decade of life, increasing with advancing age [19, 21, 28]. However, hearing loss in younger patients with OI is not uncommon [17, 22], and mixed or sensorineural hearing loss may be observed at any age [17]. When sensorineural hearing loss is found in children, it is typically found in type I OI [17, 18]. Other investigators have reported that hearing loss in OI is typically sensorineural in origin [29] and begins with a characteristic loss in the 6000–8000 Hz frequency region. One recent study reported a prevalence of sensorineural hearing loss in 12% of ears, conductive hearing loss in 21% of ears, and mixed hearing loss in 29% of ears [18] in patients with OI. As has been reported in other investigations [17, 22], sensorineural hearing loss was present in a younger age group consisting of children (mean age = 9.87) and was found only in association with type I OI [18]. 

 The etiology of sensorineural hearing loss in OI has not been definitively determined but may be a consequence of atrophy of the cochlear hair cells and the stria vascularis as well as from abnormal bone formation in the cochlea and surrounding structures [25]. Patients with OI are at greater risk for skull fractures which are associated with hearing loss. The cause of conductive impairments in OI is often associated with footplate fixation [25] although fractures of the crura do occur [30]. Atrophy of the stapes crura and hypervascularized mucosa have also been reported [25]. An association between conductive hearing loss due to stapes fixation and a mutation in the COL1A1 gene has been suggested [25]. On the other hand, in a much larger study, no relationship was found between the mutation types and the presence, type, severity, or age of onset of hearing loss [9]. Autopsy findings have shown hair cell loss, abnormalities of the tectorial membrane, perilymph hemorrhage, atrophy, and calcification of the stria vascularis in OI [31]. In newborns with severe OI, evidence of incomplete ossification of the otic capsule has been reported as well as microfractures in the otic capsule, deficient ossification of the ossicles, and microfractures in the anterior process and handle of the malleus [11]. In older patients with OI who had been seen surgically for stapedial fixation or discontinuity, histological examination of the recovered stapes revealed evidence of an otospongiotic-like lesion in the footplate of the stapes [32]. CT scans in OI have revealed undermineralization of the otic capsule [33] as well as involvement of the cochlea, semicircular canals, the distal internal auditory canal, and oval window in an individual with hearing loss of mixed origin [33]. It has been noted that in some respects the features of hearing loss in OI such as ossicular involvement and hearing loss progression mimic those found in otosclerosis [21]. However, OI and otosclerosis are biochemically divergent [34]. Examination of histopathological data has shown that OI is a generalized disorder of the bone and connective tissue, whereas otosclerosis is a localized disease of the temporal bone [32]. Another difference between OI and otosclerosis is that sensorineural hearing loss is more commonly reported in OI than in otosclerosis and at earlier ages [17]. 

Hearing loss is very common in older patients with OI and in one recent study in older patients (Mean age = 44 years) with OI, 87% of ears had some degree of hearing loss [18]. In younger patients with OI, hearing loss was not as common but was still present in 38% of ears [18]. Other studies with pediatric patients with OI have reported prevalence rates ranging from 9.1% [35] to 77.3% [22]. When patients with a range of medical conditions including otitis media with effusion, skull fracture, and neonatal meningitis are included in the sample studied, higher prevalence rates are reported [22]. Some investigators [18, 22] have not made assumptions as to whether factors present in the medical history were related or not to the complex of symptoms experienced by patients with OI. For example, the presence of OME, history of head trauma, and bacterial meningitis were not exclusion criteria in recent investigations as long as the patients presented with the diagnosis of OI [18, 22]. This practice has been challenged by some investigators [36].

 Conductive hearing loss is typically reported as the initial manifestation of hearing loss in OI [16, 17, 19]. Clinically, pediatric patients with OI may initially present with conductive hearing loss associated with otitis media. This clinical observation has recently been supported in two investigations [18, 22], which have suggested the possibility of increased susceptibility in pediatric patients with OI to otitis media with effusion. In pediatric patients with OI, tympanometric abnormalities consistent with the presence of middle ear effusion were common in that 43% of the sample failed tympanometry [18]. In contrast, failure rates for tympanometry in preschool and school age children have been reported to be in the range of to 8.6%–13.5% [37, 38], which suggests a higher rate of failure for tympanometry in OI than in the general preschool and school age population. As noted recently [18], the high incidence of otitis media with effusion in younger patients with OI may be attributable to the presence of craniofacial dysmorphism in OI [22]. A high frequency of OME is also commonly observed in other syndromes characterized by morphological abnormalities of the temporal bone [39] or as a consequence of cranial molding or deformation [22].

As noted recently, measurement of hearing loss and the determination of etiology of the hearing loss presents some challenges in OI [18]. The use of bone conduction thresholds in determination of hearing loss etiology is potentially confounded by the presence of middle ear pathology. Immobility of the stapes footplate with consequent mechanical restriction of the perilymph may alter cochlear function sufficiently to elevate bone conduction thresholds [40]. Middle ear pathologies contributing to increased stiffness in the middle ear system impact on the ossicular inertial component of bone conduction hearing [41]. This results in poorer bone conduction thresholds. For example, bone conduction thresholds are known to be elevated in otosclerosis in the 2000 Hz region. Tympanometric abnormalities may not support the presence of conductive involvement in OI as it was recently reported that only 65% of ears with conductive hearing loss and 48% of ears with mixed hearing loss were found to have abnormal tympanograms [18]. Other investigators have reported normal tympanograms in individuals with conductive and mixed hearing loss [19]. It has been noted that it is possible that the presence of multiple otologic pathologies in OI may confuse the interpretation of tympanometric findings [18]. This is the case because pathology contributing to high admittance may be masked by pathology resulting in reduced admittance of the tympanic membrane/middle ear system [42, 43]. It has been reported that the most prevalent abnormal tympanometric types observed in young patients with OI are consistent with the presence of middle ear effusion (A_S_, B, and C) [18]. In contrast, for older patients with OI, the Type A_D_ pattern is more commonly found [18]. The type A_D_ pattern is often associated with ossicular discontinuity although it can also be consistent with atrophic scarring of the tympanic membrane. The use of only a low-frequency probe tone in these studies may explain the poor test performance of tympanometry clinically [18, 19]. The use of a two-component higher frequency (678 Hz) tympanogram has been shown to be more accurate in identification of ossicular chain disruptions than when only a 226 Hz probe tone is utilized [44].

## 3. Management of Hearing Loss in OI 

Treatment of hearing loss in OI can be divided into categories based on the severity of the hearing loss and the etiology. As noted above, the loss can be conductive (involving the ossicles, ear drum, and middle ear), sensorineural (involving the cochlea, auditory nerve, and brain), or a combination of both. The degree of hearing loss helps determine if any intervention is necessary, and if so, what that intervention should be.

Conductive hearing loss theoretically can be fully corrected. In people without OI, that is unfortunately not always possible. In people with OI, the percentages that are not correctable are higher.

**Serous Otitis Media**

Fluid in the middle ear causes loss of hearing by limiting ear drum and ossicle motion. This occurs commonly in young children but can occur at any age. Common causes include Eustachian tube dysfunction, colds, ear infections, and allergies. When treatment of the underlying cause does not resolve the fluid, ventilation tubes can be placed. The incidence of serous otitis may be higher in OI. But the treatment outcomes are the same.

**Ossicular Problems**

OI can cause deformity of the ossicles described earlier. These deformities can lead to a conductive hearing loss from ossicular fracture and stapes footplate fixation. Surgical correction is usually possible, but results of such surgery are significantly worse than similar surgery in non-OI series reports (Table 2). Stapedectomy results in type I OI show closure of the air-bone gap to within 10 db in 75–85% compared to published results in normal patients of 90–95%. Postoperative hearing loss is also higher in OI patients with up to 8% losing hearing instead of the 1% seen in the non-OI group. The reasons for the failure are easily understood. In stapes surgery, the footplate may be very thick and highly vascular. This limits visibility and may narrow the distance between the footplate and the inner ear balance organs (utricle and saccule) to less than 0.5 mm. Also in all reconstructions including stapedectomy, the supporting bone being used is weaker than and thus not as stable as non-OI bone.

Treatment for sensorineural hearing loss in people with OI is similar to people without OI. Hearing aids can help manage sensorineural, conductive, and mixed (both sensorineural and conductive) hearing loss. Since hearing aids amplify the sound going into the ear, the amount of the loss is more important than the cause of the loss. However, it should be noted that individuals with sensorineural hearing loss typically have greater problems with speech recognition and loudness processing than is the case with conductive hearing loss. Fitting a person with OI with a hearing aid is the same as fitting someone without OI.

**Cochlear Implants**

2–11% of patients with OI will have hearing deterioration such that hearing aids will no longer be useful. Cochlear implants provide an option for restoring hearing [45–47]. They are surgically implanted into the inner ear and electrically stimulate the auditory nerve directly. Some problems have been encountered that are specific to people with OI. The operation to implant the device can be more difficult because of the hypervascularity of the bone and the possible narrowing of the inner dimensions of the cochlear channel. Also, once the device is placed, the bone does not shield the electrical stimulating current as well because of the lower bone density. This can sometimes lead to stimulation of the facial nerve and require some of the electrodes to be turned off. Despite these limitations, cochlear implants provide great benefit in deaf individuals with OI.

**Bone-anchored hearing aids (BAHA)**

This is an alternative treatment for conductive hearing loss and single-sided deafness. It requires one normal functioning inner ear. It also requires that the titanium implant osseointegrates into the bone. A hearing aid is then attached to the implant to directly stimulate the bone. This vibration is sent to the inner ear to stimulate hearing by bypassing the ossicles. This is the same integration technology used with some dental implants. Since many patients with OI develop bilateral sensorineural hearing loss, they may not be a candidate for the BAHA anyway. With the high percentage of people with sensorineural hearing loss, the number of qualified BAHA candidates will be low. To date, no data on the use of this implant in OI is available.

Implantable hearing aids are starting to make their way into the option list for treating hearing loss. These devices drive the ossicular chain directly rather than amplifing the sound through air. They are attached to the ossicles surgically. Given the fragility of the ossicles in OI, they may not be a good option. However, beneficial results of Vibrant SoundBridge implantation in combination with stapedectomy have recently been reported in a small sample of three patients with OI [48].

## Figures and Tables

**Table 1:** Types of osteogenesis imperfecta (adapted from [18]).

Type	Inheritance**	Clinical	Incidence^++^	Mutations
I	AD	Mild, blue sclerae fractures with little or no deformity hearing loss, DI	60%	Type I collagen COL1A1, COL1A2
II	AD, AR	Lethal, pulmonary insufficiency, beaded ribs, rhizomelic hearing loss	10%	Type I collagen COL1A1, COL1A2
III	AD, AR	Progressive deforming intrauterine fractures, deformed limbs, scoliosis white or blue sclerae hearing loss, DI	10%	Type I collagen COL1A1, COL1A2
IV	AD	Moderately severe, limb deformity, sclerae blue early and lighten with age scoliosis	15%	Type I collagen COL1A1, COL1A2
V	AD	Variable phenotype like IV hyperplastic callus, dislocated radial head calcified interosseous membrane	5%	Unknown
VI	unknown	More fractures than IV mineralization defect on biopsy, vertebral fractures, no DI	Unknown	Type I collagen SERPINF1 (chaperone protein)
VII	AR	First nations family, Quebec	^++^	CRTAP, LEPRE1, PPIB (prolyl-3 hydroxylation)
		congenital fractures white sclerae, severe rhizomelia		
VIII	AR	Severe or lethal similar to OI type II (Sillence)	^++^	CRTAP, LEPRE1 SERPINH1

**AD = autosomal dominant.

**AR = autosomal recessive.

^++^: the incidence of OI types I–IV is reasonably established. However, for the less common types, OI types VI, VII, and VIII, the incidence is not clearly defined. However, it is estimated that the recessively inherited forms (VII and VIII) constitute approximately 3–5% of the total OI population.

**Table 2:** Results of stapedectomy in people with and without OI. Findings depict the number of cases, percent of patients with postoperation air-bone gap of less than 10 dB HL, and percent of patients with postoperation hearing loss.

	Cases	Post-op air bone gap <10 dB	Post-op sensorineural hearing loss
OI series			
Garretsen and Cremers [49]	40	78	1.7
Shea and Postma [50]	51	75	8
Ferekidis et al.[51]	9	75	0
Vincent et al.[52]	21	85.7	0

Non-OI series			
Vincent et al. [53]	2525	94.2	0.7
